# Organizing national responses for rare blood disorders: the Italian experience with sickle cell disease in childhood

**DOI:** 10.1186/1750-1172-8-169

**Published:** 2013-10-20

**Authors:** Raffaella Colombatti, Silverio Perrotta, Piera Samperi, Maddalena Casale, Nicoletta Masera, Giovanni Palazzi, Laura Sainati, Giovanna Russo

**Affiliations:** 1 Clinica di Onco-Ematologia Pediatrica, Dipartimento della Salute della Donna e del Bambino, Azienda Ospedaliera-Università di Padova, Via Giustiniani 3, 35128, Padova, Italy; 2 Dipartimento della Donna, del Bambino e della Chirurgia Generale e Specialistica, Seconda Università di Napoli, Via L. De Crecchio, 2, 80138, Napoli, Italia; 3 Unità di Ematologia ed Oncologia Pediatrica, Dipartimento di Pediatria, Università di Catania, Via Santa Sofia 78, Catania 95123, Italy; 4 Dipartimento di Pediatria, Università di Modena e Reggio Emilia, via del Pozzo 71, 41100, Modena, Italy; 5 Dipartimento di Pediatria, Ospedale San Gerardo, Via Pergolesi 33, 20052, Monza, Italy

**Keywords:** Sickle cell disease, Child, Rare blood disorder, Health education

## Abstract

**Background:**

Sickle cell disease (SCD) is the most frequent hemoglobinopathy worldwide but remains a rare blood disorder in most western countries. Recommendations for standard of care have been produced in the United States, the United Kingdom and France, where this disease is relatively frequent because of earlier immigration from Africa. These recommendations have changed the clinical course of SCD but can be difficult to apply in other contexts. The Italian Association of Pediatric Hematology Oncology (AIEOP) decided to develop a common national response to the rising number of SCD patients in Italy with the following objectives: 1) to create a national working group focused on pediatric SCD, and 2) to develop tailored guidelines for the management of SCD that could be accessed and practiced by those involved in the care of children with SCD in Italy.

**Methods:**

Guidelines, adapted to the Italian social context and health system, were developed by 22 pediatric hematologists representing 54 AIEOP centers across Italy. The group met five times for a total of 128 hours in 22 months; documents and opinions were circulated via web.

**Results:**

Recommendations regarding the prevention and treatment of the most relevant complications of SCD in childhood adapted to the Italian context and health system were produced.

For each topic, a pathway of diagnosis and care is detailed, and a selection of health management issues crucial to Italy or different from other countries is described (i.e., use of alternatives for infection prophylaxis because of the lack of oral penicillin in Italy).

**Conclusions:**

Creating a network of physicians involved in the day-to-day care of children with SCD is feasible in a country where it remains rare. Providing hematologists, primary and secondary care physicians, and caregivers across the country with web-based guidelines for the management of SCD tailored to the Italian context is the first step in building a sustainable response to a rare but emerging childhood blood disorder and in implementing the World Health Organization’s suggestion “to design (and) implement … comprehensive national integrated programs for the prevention and management of SCD".

## Background

Sickle cell disease (SCD) is the most common genetic disorder worldwide. Every year, an average of 300,000 children are born with sickle syndromes, and the prevalence of sickle cell among newborns ranges from 0.1/1000 in non-endemic countries to 20/1000 in several parts of Africa [1]. The United Nations and the World Health Organization (WHO) recognize inherited hemoglobin (Hb) disorders as a global public health problem [2,3], and the WHO has urged the National Health Systems “to design, implement and reinforce … comprehensive national integrated programs for the prevention and management of SCD” [2]. In fact, although 19–27% of the African population carries the sickle cell allele, migration movements have brought SCD patients from high-frequency regions to Europe. Therefore, SCD has become the paradigm of immigration hematology in Europe [4]. Although considered a rare disease because of its global frequency in the 28 countries of the European Union, SCD is the most prevalent genetic disease in France [5] and the United Kingdom [6], and its frequency is steadily rising in many other countries of central and southern Europe [4,7,8]. The European Union considers SCD a rare disease and sponsored the European Network on Rare and Congenital Anemias (known as ENERCA) to facilitate collaboration among clinicians in the field of rare anemias, including SCD [9]. Several countries have developed a specific response to meet the needs of the growing number of patients with SCD. In France, national guidelines have been produced [10] and reference centers have been designated by the Health Ministry that offer focused services for SCD [11]; a newborn screening was performed at national level since 2000 for all newborns defined as being “at risk” for SCD based on ethnic origin [12]. In the United Kingdom, the development of national guidelines and the realization of a universal newborn screening program have been the first steps undertaken to offer specialized care [6]. Belgium and the Netherlands have also organized clinical services and newborn screening for hemoglobinopathies and are conducting careful investigations [13,14].

Although the experience gained from the United States and the above mentioned European countries has forged a pathway for those involved in the care of patients with SCD, public health issues and policies associated with SCD need to be tailored according to each country’s availability of health care and the characteristics of the affected population [15-17]. Italy does not have a national hemoglobinopathy newborn screening program, and for many years, pediatric hematology services have focused primarily on beta thalassemia. A network for treatment and care of thalassemia patients has been developed in Italy since the 1970s, involving centers in the central and southern parts of the country, where the majority of thalassemia patients live. Moreover, guidelines for the management of thalassemia were implemented and are well known by physicians across the country. National public health interventions for prevention and screening of thalassemia conditions are widespread; sound research has been conducted, and patient associations continuously raise awareness of thalassemia and advocate for support.

In contrast, SCD was historically a disease limited to the population of Sicily where the sickle cell allele frequency is 2–13% [18]. Recent immigration from Africa, South America, and the Balkans, mainly into the northern regions, has changed the geographic profile of SCD [7,19,20], however, with an increase of new diagnoses in the north, making this disease a more widespread public health issue [21]. Although the number of affected children has steadily increased over the last decade [7,19-21], by the end of 2008, centers focused on the care of SCD were still rare, and clinical counseling for SCD was performed either by hematologists trained for thalassemia and not for SCD, or by pediatricians. No common policy of diagnosis and treatment had been developed, and the treatment was mainly case directed or locally directed. Moreover, the majority of SCD patients were African immigrants, living in northern Italy where hemoglobinopathies were less known.

The Italian Association of Pediatric Hematology Oncology (AIEOP) has a membership of all 54 Pediatric Hematology Oncology Centers across Italy and has existed since the 1970s (the website is http://www.aieop.org). It has developed common protocols and a database for children with cancer and non-oncologic diseases. The AIEOP Red Cell Group, involved in non-oncologic hematology, decided to develop a common national response to the rising number of SCD patients. Its objectives are creating a national working group focused on pediatric SCD and developing tailored guidelines for the management of children with SCD in Italy that could be accessed and practiced by all those involved in the care of the pediatric SCD population in the country.

We thought that creating a network of physicians involved in the day-to-day care of children with SCD and providing physicians and caregivers across the country with guidelines for the management of SCD tailored to our specific Italian context were the first steps in building a sustainable response to this newly more widespread disorder. The purpose of this paper is not to detail the guidelines but to describe the pathway chosen by the AIEOP Group to address a rare but increasingly prevalent blood disorder in Italy and summarize the recommendations that were critical for our specific health system and national context.

## Methods

All 54 AIEOP centers were invited to participate in the SCD Working Group. Issues to be addressed in the guidelines were chosen by the group considering the international recommendations and the most important clinical issues in pediatric SCD [6,22,23]; every topic was developed by a subgroup in a single document. Each document included a brief description of the state-of-the-art recommendations with ABC ranking of strength of the evidence and references. Statements were based on literature evidence; when available evidence was not sufficient, the working group developed specific recommendations, based on expert author opinion or existing guidelines, and such statements were clearly presented as opinion. Each document was revised by the entire group and modified accordingly after exhaustive discussion. Four members of the group completed the internal revision of the final document, and external revision was performed by three Italian expert pediatric hematologists and one patient association.

## Results and discussion

The SCD working group consisted of 22 pediatric hematologists from 14 centers (north 9, central 1, south 4), who met five times during 20 months from 2008 to 2010, for a total of 120 hours of meetings. One final 8-hour meeting was held in 2012 for definitive revision and approval of the document. Specific characteristics of our health system were taken into consideration in writing the guidelines. (see below List of Characteristics of the Italian context).

Characteristics of the Italian context

No national hemoglobinopathies newborn screening program

National free health care; direct primary, secondary, and tertiary pediatric care for children

Regulation of health care provided by regional health care systems with regional differences and disparities in health care facilities and resources across the country

Specialized care delivered on a hospital-based model and not on a medical home-based model

Free vaccinations with vaccination campaigns conducted on regional basis

Care for sickle cell patients delivered in pediatric hematology oncology centers among oncology patients

→ lack of teams dedicated to sickle cell patients

→ management of pain crisis in the emergency room

No availability of oral penicillin in the country

No availability of pediatric transcranial Doppler services, but an adult stroke network exists in the country

### Recommendations

The guidelines, written in Italian, consist of 27 chapters and 242 recommendations and explore the milestones of SCD diagnosis, prevention, and treatment, tailoring them to the Italian public health system organization and drug availability. Recommendations that were considered more critical in our context and deserved extensive discussion are described below.

#### Neonatal screening

Universal neonatal screening, or at least targeted screening, is recommended. A comprehensive program for global *prix en charge* should enroll children after the neonatal diagnosis, with adequate health education for parents, implementation of preventive and therapeutic measures, and appropriate follow-up. We are aware that for the present, only two pilot selective screenings are being conducted in Italy, organized on a local basis (in the Region of Friuli Venezia Giulia and in the city of Modena).

#### Amoxicillin prophylaxis and vaccinations

Since its introduction for children with SCD, penicillin prophylaxis has resulted in a large reduction of invasive pneumococcal infections; therefore, it is recommended starting at 2 months of age (Table 1). In Italy, the oral formulation of penicillin is not available, and a depot i.m. formulation is difficult to find; therefore, the group made an effort to search the literature and to elaborate alternative prophylaxis options. The recommendation for Italy is to use amoxicillin or, as an alternative, a macrolide (Table 2). Pneumococcus immunization with both conjugated (PVC13) and polysaccharidic (Pneumo23) vaccine is recommended. Annual influenza vaccination is also recommended to reduce possible severe complications [24].

**Table 1:** Recommendation for penicillin prophylaxis

**Recommendation**	**Evidence**
Penicillin prophylaxis is strongly recommended for all children with SCD (homozygous SS, SC disease, Sβ °thalassemia) up to 6 years of age.	A
Penicillin prophylaxis is recommended in subjects with genotype SC, even if no clear study demonstrates its benefit in this form of SCD.	C
Prophylaxis with oral penicillin should be done, but because it is not available in Italy, the alternative drugs described in Table 3 or the parenteral formulation can be used.	C
Antibiotic prophylaxis should begin between the second and third months of life in children who received a neonatal diagnosis of SCD.	A
In Italy, newborn screening is not provided; therefore, the pediatrician who cares for an infant at high risk of SCD (geographic origin, family history of hemoglobinopathy) should test the infant for the presence of the disease, even in the absence of symptoms, and prescribe prophylactic penicillin by the third month.	B
It is controversial whether it is necessary to continue penicillin prophylaxis beyond 5 years of age, although it is certainly more prudent to recommend the continuation of prophylaxis throughout life.	C
Long-term prophylactic therapy causes problems of adherence, which may be limited by clear information about the benefits of prophylaxis, the risks of a low adherence, and the involvement of parents in the management of disease and care of the child.	C

**Table 2:** Alternative drugs for antibiotic prophylaxis

**Proposing group**	**Age**	**Recommendation for antibiotic prophylaxis**		
		**Antibiotic**	**Dose**	**Frequency**
Working Party of the British Committee for	<5 y	Amoxicillin	10 mg/kg/d	Once a day
Standards in Haematology Clinical	5–14 y	Amoxicillin	125 mg/d	Once a day
Haematology Task Force [25,26]	>14 y	Amoxicillin	250–500 mg/d	Once a day
The Hospital for Sick Children, Toronto [27-29]	2–6 months	Trimethoprim/sulfamethoxazole	TMP 5 mg SMX 25 mg/kg	Once a day
	6 months–5 y	Amoxicillin	20 mg/kg/d	Twice a day
	>5 y	Amoxicillin	250 mg/day	Twice a day
Australasian Society for Infectious Diseases [30]	2 months–2 y	Amoxicillin	20 mg/kg/d	Once a day
			(max 250 mg/d)	Once a day
	Adults	Amoxicillin	250 mg/d	

#### Management of painful vaso-occlusive crisis (VOC)

VOCs are the hallmark of SCD, and extensive literature has demonstrated that the optimal management of VOC is in hematology day care centers [31,32]. In Italy, SCD patients with VOC are usually admitted to the general emergency room (ER), however; therefore, the guidelines emphasize the need to train ER personnel in the management of VOC. Generally, pediatricians are not comfortable with the use of analgesics, especially opioids; consequently, the guidelines detail each drug, including the safety profile and practical dilution modality, to make their use easier even for pediatricians who are not familiar with them. Table 3 lists the differential diagnoses of VOC and acute emergencies to ease patient care for ER personnel who are not familiar with SCD patients.

**Table 3:** Differential diagnosis between vaso-occlusive crisis and other acute emergencies

**Site of pain**	**Differential diagnosis**	**Exams to consider**
Head	Hemorrhagic stroke	MRI, MRA
	Sinusitis	CT
	Migraine/headache	Lumbar puncture
	Meningitis	
Neck/throat	Meningitis	Lumbar puncture
	Torticollis/stiff neck	Throat swab
	Pharyngitis/tonsillitis	Esophago gastro duodenal endoscopy
	Esophagitis/gastroesophageal reflux	
Thorax	Acute chest syndrome/reactive airway disease/asthma	Thorax X-ray
	Osteochondritis	ECG
	Heart (myocardial infarction)	Esophago gastro duodenal endoscopy
	Gastroesophageal reflux	
Abdomen	Acute abdomen:	Abdomen X-ray
	Appendicitis	Abdomen ultrasound
	Cholecystitis	Amylase/lipase
	Other	Thorax X-ray
	Gallbladder stones	Throat swab
	Pancreatitis	Urine exam and culture
	Splenic sequestration	
	Acute chest syndrome	
	Urinary infection/pyelonephritis	
Extremities/joints	Osteomyelitis	X-ray
	Septic arthritis	Ultrasound
	Limping without pain	MRI, MRA

A visual analogue scale (VAS) or a numerical scale is always recommended for pain evaluation. The treatment of pain is to be established as quickly as possible, within 30 minutes from arrival in the ER, even before the identification of the cause or the possible differential diagnoses. The choice of the drug/s to be used must take into account the intensity of the pain and the drugs that have been used at home. The primary objective is to reduce the initial VAS score by 50%. Pain revaluation should be performed every 30 minutes until complete resolution. Children should receive analgesics at fixed intervals with “rescue” doses for pain that occurs in the interval between doses. The interval between doses should be determined in accordance with the intensity of pain and the duration of the analgesic effect of the drug in question. Acetaminophen, ibuprofen, ketorolac, codeine, and morphine analgesics are the recommended drugs for the treatment of pain in children.

#### Management of fever and infections

The management of the febrile child was the subject of intense discussion in the working group because of the disparities in health care facilities and availability of ER staff between northern and southern Italy and because of the many isolated and rural areas lying long distances from health centers in certain parts of the country. The decision was finally to define the presence of fever in a child with SCD as a medical emergency (“All those who manage children with SCD—pediatricians, hematologists, medical emergency staff, parents—should be aware that fever is a medical emergency which must be assessed and treated in the shortest possible time”), but to suggest different pathways of diagnosis and care between children at high risk of severe infection and at standard risk of severe infection (Table 4). Children at high risk of severe infection should be admitted; children at standard risk of severe infection should receive a dose of ceftriaxone and at least 3 hours of observation. The possible active involvement of the child’s pediatrician is considered.

**Table 4:** Clinical features useful for assessing the risk of severe infection in febrile patients

	**High risk**	**Low risk**
	**(One or more of the following)**	**(All of the following)**
Age	< 3 years of age	> 3 years of age
Health conditions	Compromised	Stable
Temperature	>40°C	>38.5°C and <40°C
Refill	Increased	Normal
Hydration status	Dehydration and/or reduced fluid assumption and/or oliguria	Normal
Acute chest syndrome	Yes	No
History of sepsis or severe infection	Yes	No
Allergy to penicillin and cephalosporin	Yes	No
Blood pressure	Hypotension	Normal
Hemoglobin	<5 g/dl	<2 g/dl from baseline
White blood cells	>30 × 103/mm3 or <5 × 103/mm3	Normal
Platelets	<100 × 103/mm3	Normal

#### Acute chest syndrome (ACS)

ACS is defined as the appearance of a new infiltrate on chest X-ray in association with at least one of the following: fever, dyspnea, chest pain, or desaturation. ACS is the second leading cause of hospitalization for patients with SCD and one of the leading causes of mortality. It is a potentially severe manifestation of SCD, and the patient therefore should always be hospitalized. The useful therapeutic measures are administration of O_2_, hydration, analgesia, early transfusion, and bronchodilators (especially if there is wheezing). Antibiotic therapy is always indicated. As a first choice, the ceftriaxone (or cefotaxime) + macrolide is preferred, considering the possibility of adding an anti-staphylococcal antibiotic. The use of “incentive spirometry” is indicated and is also effective in preventing the onset of ACS in all conditions of reduced thoracic mobility (chest pain crisis, immobilization in bed, etc.). There should be a timely transfer to the Intensive Care Unit (ICU) with sudden clinical worsening, requiring ventilatory support. The use of steroids is at present controversial, and the use of nitric oxide is still experimental. Because of the increasing recognition of this condition as a risk factor of disease severity in SCD, wheezing and asthma must be recognized and treated, as well as allergies for wheezing prevention.

#### Transcranial Doppler (TCD)

Early TCD screening and transfusion programs applied in patients with abnormal TCD really allows to significantly reduce stroke risk from 11 to less than 2% by age 18 and should be systematically offered to all children with SCD [33-35].

Because of the deficiency of this procedure in the Italian context, a particular emphasis has been given to this chapter, highlighting the difficulties other countries have faced in setting up a TCD program (lack of trained radiologists, missed appointments, education of patients and their parents, training of providers-pediatricians, hematologists, pediatric radiologists, service organization and patients’ access). In fact, in the USA, despite incontrovertible evidence of the value of screening for stroke risk, the adherence to the TCD surveillance programs is still less than 50% [36]. The results of a nationwide survey revealed poor compliance because of several obstacles, with the most frequent problems being poor patient attendance (as a result of the distance to the vascular screening laboratory) and the lack of personnel trained to perform TCD. The large majority of surveyed hematologists experienced difficulty in obtaining TCD assessment for their patients [37]. McCarville et al. [38] highlighted the importance of a comprehensive screening program in a single-center study in which the center achieved a 99% screening rate of the at-risk population in 3 years. The success of this comprehensive program relied on investing resources in personnel training and patient education.

TCD is recommended in children with SCD from ages 2 to 16 at least once a year It must be performed according to a specific protocol by trained staff to obtain reliable and reproducible results. If TCD is normal, a repeat screen is recommended after 12 months; if it is between 170–200 cm/s (conditional), it should be repeated within 3 months, especially if the child is under age 6 years. In individuals with TCD > 200 cm/s, a second test should be repeated within 2 months and, if confirmed as pathological, enrollment in a chronic transfusion program is recommended. In patients with abnormal TCD, another recommendation is to perform an MRI and MRA investigation for possible detection of brain injury. If blood flow is too low (TCD < 70 cm/s) or there is significant asymmetry between the two sides or if TCD cannot be performed, it is necessary to complete alternative neuroimaging studies, such as MRI and MRA. TCD imaging can be used in place of the TCD.

#### Blood transfusion

The issue of blood transfusion has been particularly focused to clearly differentiate indications for transfusion in SCD from thalassemia; the risk of alloimmunization has been stressed because of the lack of African donors in Italy and the need to avoid alloimmunization as much as possible (Table 5).

**Table 5:** Recommendations to decrease the risk of alloimmunization

**Recommendations**	**Evidence**
Perform a wide erythrocyte antigenic phenotype (ABO, Rh, Kell, Duffy, Kidd, Lewis, Lutheran, P, and MNS) before the first transfusion, especially if you plan to establish a chronic transfusion program; physicians, health care providers, and the patient or family should have a copy of the same phenotype.	C
Pre-storage leukodepletion of RBCs is recommended to reduce febrile reactions and complications due to cytokine release.	C
All patients who have previously performed red cell transfusions should be periodically checked for alloantibodies (they can cause a delayed transfusion reaction).	C
Preferably use “fresh” blood (<3 days of life of RBCs) to minimize hypoxia during the procedure and to reduce the consumption of RBCs in chronically transfused patients while minimizing iron overload.	C
Use blood negative for hemoglobinopathies. Each center must activate a strategy suited to avoid transfusing blood of carriers of Hemoglobin S.	C

Indications for acute transfusion are acute symptomatic anemia with clinical signs of heart failure (tachycardia, tachypnea, dyspnea, fatigue) or absolute Hb ≤ 5 g/dl; it should be considered for a drop in Hb ≥ 2 g/dl from steady state or acute hepatic or splenic sequestration [1,6]. In this last case, the values of Hb measured after transfusion are generally higher than expected; therefore, to prevent heart failure, it is better to transfuse 3.5 ml/kg of packed red blood cells, equal to half the usual dose [1] and then repeat the transfusion after a few hours. Great care must always be taken to avoid exceeding 10 g/dl Hb, above which the phenomenon of hyperviscosity may be dangerous. In severe infection, sepsis, or meningitis with significant anemia (Hb < 7 g/dl), blood transfusion should be performed because of the lower tolerance to anemia in the course of severe infection. In cases of high levels of Hb and transfusion indication, as in ACS or stroke, erythrocytapheresis is mandatory.

The indications for chronic transfusion are primary prevention of stroke in children with abnormal TCD, secondary prevention of stroke, and chronic heart failure, although there are no clinical studies that have shown its effectiveness in children. The goal of a chronic transfusion program is to maintain HbS level under 30% and Hb between 9 and 11 g/dl.

#### Iron overload and iron chelation

Iron overload has been a widely discussed topic, considering the lack of any official indication about its monitoring and treatment in SCD. Differences between thalassemia and SCD in the pattern of iron accumulation and complication rate have been the focus [39-43], and indications for monitoring, start of treatment, and drug choice have been addressed. Parameters for monitoring of iron overload were considered and specific recommendations made (Table 6). Recommended criteria for deciding the start of iron chelation therapy are serum ferritin level persistently ≥ 1000 ng/ml and transfused red blood cells ≥ 120 cc/kg (or after 20 transfused units) and/or a Liver Iron Concentration (LIC) ≥ 5–7 mg/g dw.

**Table 6:** Recommendations for monitoring iron overload

**Recommendations**	**Evidence**
Serum ferritin level could increase during VOC; assessment at steady state is recommended.	C
Serial measurements of serum ferritin and iron intake are recommended before starting iron chelation therapy.	C
LIC (liver iron concentration), measured by magnetic resonance T2* or R2*, SQUID (superconducting quantum interference device), or MID (magnetic iron detector), all techniques available in Italy, should be assessed before starting iron chelation therapy.	C
Cardiac iron assessment, measured by magnetic resonance T2*, is recommended before starting iron chelation therapy.	C

Compliance with chelation therapy affects morbidity and mortality in iron overloaded patients [44,45], so a drug must be evaluated considering patient satisfaction, convenience, activity limitations, and preferences. Deferoxamine is an effective drug with an acceptable safety profile [46,47], but patient adherence to this therapy is poor because of side effects and difficult route of administration (subcutaneous infusions over an 8- to 12-hour period, 5–7 days per week). Deferasirox is an oral chelator that has shown efficacy and safety in managing iron overload in SCD [48]. Patients on deferasirox have higher satisfaction and rate its convenience higher than patients do for deferoxamine [49], so deferasirox is rapidly becoming the standard care.

#### Hydroxyurea (HU)

Because of the reluctance of pediatricians to prescribe HU, specific indications and the safety profile have been particularly stressed in the guidelines. The clinical efficacy and low toxicity of HU have been demonstrated by the multi-center study HUG-KIDS Phase I/II [50], and recently in the randomized, double-blind, BABYHUG, which demonstrated the safety and efficacy of HU in “infants” (mean age 13.6 months) in reducing episodes of dactylitis, pain, and ACS in a population of patients not selected according to the clinical severity of the disease. The study also confirmed the safety of administration in the first few years of life [51]. Pediatric studies have highlighted other potential benefits of HU in the prevention of organ damage, the maintenance of splenic function, and improved growth [52,53]. It is still unclear what role HU can have in primary and secondary prevention of stroke.

### Conclusion and implications for public health practice

The organization of a national network and the development of national guidelines for pediatric SCD in Italy are the first steps in implementing the WHO suggestion “to design (and) implement … comprehensive national integrated programs for the prevention and management of SCD” and to address the two major priorities for the treatment of SCD, identified by patients in a questionnaire submitted in 2007 by the National Heart Lung and Blood Institute. One top priority that patients identified was the development of clear, evidence-based, local guidelines that they and their physicians could use to ensure that they were receiving optimal care, maximizing appropriate referral to hematologic specialist [54]. The second priority was appropriate management of pain. The development of national guidelines targeted to a specific national context allows addressing both priorities. The organization of the national network and the diffusion of the locally adapted guidelines in Italy were followed by several educational events and training courses on the management of SCD in childhood and TCD that involved hundreds of primary and secondary care pediatricians and pediatric hematologists across the country. Patient empowerment initiatives involving immigrant communities have begun to be developed, and every year the SCD World Day is now celebrated in most centers with open initiatives.

A better and standardized management of this relatively rare but complex disease implies that health systems will waste less money to treat acute or chronic complications and will optimize patient management. Children with SCD are generally admitted to ER and hospitals much more than their peers [7,16,17]. Moreover, SCD is in fifth place among the top 10 diagnoses with the highest readmission frequency, having the highest percentage of readmission (79.4%) [55]. The yearly cost of care for a pediatric patient 1–9 years of age with SCD is 7948 euro in countries where neonatal screening allows early diagnosis and comprehensive care is organized, assuring a holistic approach to health care; the morbidity and mortality and, therefore, the costs are much higher in cases of late diagnosis or no access to SCD specialized centers [16,17,56-60]. A preliminary single-center experience even in a low-prevalence country such as Italy has shown that comprehensive specialized care for a vulnerable group of immigrant children with SCD produces lower admission rates and lower use of the ER [61]. Detailed cost analysis is available for the USA but scarce for European countries; therefore, research in this field in Europe is needed.

In conclusion, creating a network of physicians involved with the day-to-day care of children with SCD and providing physicians and caregivers across the country with guidelines for the management of SCD tailored to the specific Italian context can be the first steps in building a sustainable response to a rare but nationally emerging blood disorder in childhood.

## Competing interests

The authors declare that they have no competing interests.

## Authors informations

Members of the *AIEOP Sickle Cell Disease Working Group*

Andrea Ciliberti (San Giovanni Rotondo, Italy), Gian Carlo Del Vecchio (Bari, Italy), Domenico De Mattia (Bari, Italy), Benedetta Fabrizzi (Ancona, Italy), Cinzia Favara Scacco (Catania, Italy), Paola Giordano (Bari, Italy), Valentina Kiren (Triseste, Italy), Saverio Ladogana (San Giovanni Rotondo, Italy), Agostino Nocerino (Udine, Italy), Lucia Dora Notarangelo (Brescia, Italy), Anna Pusiol (Udine, Italy), Anita Regalia (Monza, Italy), Paola Saracco (Torino, Italy), Marco Zecca (Pavia, Italy).

## Authors’ contributions

RC, SP, PS, MC, GP, NM, LS, GR participated in the meetings and wrote the guidelines; RC, PS, LS and GR internally reviewed the guidelines; RC, SP drafted the manuscript; LS and GR reviewed the manuscript. All authors have seen and approved the manuscript.

## References

[B1] Weatherall DJ Clegg JB Inherited haemoglobin disorders: an increasing global health problem Bull World Health Organ 2001 79 704 712 11545326 PMC2566499

[B2] WHO Report A59/9 on Sickle Cell Anemia 2006 Available from: http://www.who.int/gb/ebwha/pdf_files/WHA59-REC1/e/WHA59_2006_REC1-en.pdf

[B3] UN Resolution A/63/L.63 “Recognition of Sickle-Cell Anaemia as a Public Health Problem” 2008 Available from: http://www.un.org/News/Press/docs/2008/ga10803.doc.htm

[B4] Roberts I de Montalambert M Sickle Cell disease as a paradigm of immigration haematology: new challenges for immigration hematologists in Europe Haematologica 2007 92 865 871 10.3324/haematol.11474 17606434

[B5] de Montalembert M Girot R Galactéros F Sickle cell disease in France in 2006: results and challenges Arch Pediatr 2006 13 1191 1194 10.1016/j.arcped.2006.06.006 16824741

[B6] NHS Standard and Guidelines for Clinical Carehttp://www.sct.screening.nhs.uk/standardsandguidelines

[B7] Colombatti R Dalla Pozza LV Mazzucato M Sainati L Pierobon M Facchin P Hospitalization of children with sickle cell disease in a region with increasing immigration rates Haematologica 2008 93 463 464 10.3324/haematol.11766 18310539

[B8] Mañú Pereira M Corrons JL Neonatal haemoglobinopathy screening in Spain J Clin Pathol 2009 62 22 25 10.1136/jcp.2008.058834 19103853

[B9] European Network on Rare and Congenital Anemiashttp://www.enerca.org/

[B10] http://www.has-sante.fr/portail/upload/docs/application/pdf/Drepanocytose_reco.pdf. Last accessed October 10th 2013

[B11] Plan National Maladies Rares 2005-2008 Assurer l’équité pour l’accès au diagnostic, au traitement et à la prise en charge http://asso.orpha.net/ORPHAWEB/cgi-bin/file/Plan_Maladies_Rares.pdf. Last accessed October 10th 2013.

[B12] Bardakdjian-Michau J Bahuau M Hurtrel D Godart C Riou J Mathis M Goossens M Badens C Ducrocq R Elion J Perini JM Neonatal screening for sickle cell disease in France J Clin Pathol 2009 62 31 33 10.1136/jcp.2008.058867 19103855

[B13] Gulbis B Cotton F Ferster A Ketelslegers O Dresse MF Rongé-Collard E Minon JM Lé PQ Vertongen F Neonatal haemoglobinopathy screening in Belgium J Clin Pathol 2009 62 49 52 10.1136/jcp.2008.060517 19103861

[B14] Bouva MJ Mohrmann K Brinkman HB Kemper-Proper EA Elvers B Loeber JG Verheul FE Giordano PC Implementing neonatal screening for haemoglobinopathies in the Netherlands J Med Screen 2010 17 58 65 10.1258/jms.2010.009075 20660432

[B15] Raphael JL Kavanagh PL Wang CJ Mueller BU Zuckerman B Translating scientific advances to improved outcomes for children with sickle cell disease: a timely opportunity Pediatr Blood Cancer 2011 56 1005 1008 10.1002/pbc.23059 21488152

[B16] Olney RS Preventing morbidity and mortality from sickle cell disease. A public health perspective Am J Prev Med 1999 16 116 121 10.1016/S0749-3797(98)00140-8 10343888

[B17] Grosse SD James AH Lloyd-Puryear MA Atrash HK A public health framework for rare blood disorders Am J Prev Med 2011 41 Suppl 4 S319 S323 22099353 10.1016/j.amepre.2011.09.010

[B18] Schiliro G Sicily: the world reservoir for thalassemias and haemoglobinopathies Nature 1978 276 761 10.1038/276761a0 723952

[B19] Russo-Mancuso G Romeo MA Guardabasso V Schilirò G Survey of sickle cell disease in Italy Haematologica 1998 83 875 881 9830795

[B20] Russo-Mancuso G La Spina M Schiliro G The changing pattern of sickle cell disease in Italy Eur J Epidemiol 2003 18 923 924 10.1023/A:1025635118305 14561055

[B21] Cataldo F Immigration and changes in the epidemiology of hemoglobin disorders in Italy : an emerging public health burden Ital J Pediatr 2012 38 32 10.1186/1824-7288-38-32 22823956 PMC3480959

[B22] American Academy of Pediatrics Health supervision for children with sickle cell disease Pediatrics 2002 109 526 535 11875155 10.1542/peds.109.3.526

[B23] National Institute of Health, National Heart, Lung and Blood Institute The Management of Sickle Cell Diseasehttp://www.nhlbi.nih.gov/health/prof/blood/sickle/sc_mngt.pdf

[B24] Colombatti R Perrotta S Masera N Palazzi G Notarangelo LD Pusiol A Bonetto E De Zen L Nocerino A Samperi P Russo-Mancuso G Sainati L Lessons learned from the H1N1 pandemic: the need to improve systematic vaccination in sickle cell disease children. A multi Center survey in Italy Vaccine 2011 29 1126 1128 10.1016/j.vaccine.2010.11.089 21147126

[B25] Working Party of the British Committee for Standards in Haematology Clinical Haematology Task Force Guidelines for the prevention and the treatment of infection in patients with an absent or dysfunctional spleen BMJ 1996 312 430 434 8601117 10.1136/bmj.312.7028.430 PMC2350106

[B26] Davies JM Barnes R Milligan D Update of guidelines for the prevention and treatment of infection in patients with an absent or dysfunctional spleen Clin Med 2002 2 440 443 10.7861/clinmedicine.2-5-440 12448592 PMC4953085

[B27] Price VE Dutta S Blanchette VS Butchart S Kirby M Langer JC Ford-Jones EL The prevention and treatment of bacterial infections in children with asplenia or hyposplenia: practice considerations at the hospital for sick children, Toronto Pediatr Blood Cancer 2006 46 5 597 603 10.1002/pbc.20477 16333816

[B28] Price VE Blanchette VS Ford-Jones EL The prevention and management of infections in children with asplenia or hyposplenia Infect Dis Clin N Am 2007 697 710 10.1016/j.idc.2007.07.002 17826619

[B29] Infectious Diseases and Immunization Committee Cps Prevention and therapy of bacterial infections foe children with asplenia or hyposplenia Paediatr Child Health 1999 4 417 421 20212952 10.1093/pch/4.6.417 PMC2827744

[B30] Spelman D Buttery J Daley A Isaacs D Jennens I Kakakios A Lawrence R Roberts S Torda A Watson DA Woolley I Anderson T Street A Australasian society for infectious diseases: guidelines for the prevention of sepsis in asplenic and hyposplenic patients Int Med J 2008 38 349 356 10.1111/j.1445-5994.2007.01579.x 18284463

[B31] Benjamin LJ Swinson GI Nagel RL Sickle cell anemia day hospital: an approach for the management of uncomplicated painful crises Blood 2000 95 1130 1136 10666181

[B32] Wright J Bareford D Wright C Augustine G Olley K Musamadi L Dhanda C Knight C Day case management of sickle pain: 3 years experience in a UK sickle cell unit Br J Haematol 2004 126 87 10.1111/j.1365-2141.2004.05123.x 15352993

[B33] Adams R McKie V Nichols F Carl E Zhang DL McKie K Figueroa R Litaker M Thompson W Hess D The use of transcranial ultrasonography to predict stroke in sickle cell disease N Engl J Med 1992 326 605 610 10.1056/NEJM199202273260905 1734251

[B34] Adams RJ Brambilla D Optimizing primary stroke prevention in sickle cell anemia (STOP 2) trial investigators: discontinuing prophylactic transfusions used to prevent stroke in sickle cell disease N Engl J Med 2005 353 2769 2778 16382063 10.1056/NEJMoa050460

[B35] Bernaudin F Verlhac S Arnaud C Kamdem A Chevret S Hau I Coïc L Leveillé E Lemarchand E Lesprit E Abadie I Medejel N Madhi F Lemerle S Biscardi S Bardakdjian J Galactéros F Torres M Kuentz M Ferry C Socié G Reinert P Delacourt C Impact of early transcranial Doppler screening and intensive therapy on cerebral vasculopathy outcome in a newborn sickle cell anemia cohort Blood 2011 117 1130 1140 10.1182/blood-2010-06-293514 21068435

[B36] Raphael JL Shetty PB Liu H Mahoney DH Mueller BU A critical assessment of transcranial Doppler screening rates in a large pediatric sickle cell center: opportunities to improve healthcare quality Pediatr Blood Cancer 2008 5 647 651 18623200 10.1002/pbc.21677

[B37] Fullerton HJ Gardner M Adams RJ Lo LC Johnston SC Obstacles to primary stroke prevention in children with sickle cell disease Neurology 2006 67 1098 1099 10.1212/01.wnl.0000237398.13545.86 17000992

[B38] McCarville MB Goodin GS Fortner G Li CS Smeltzer MP Adams R Wang W Evaluation of a comprehensive transcranial doppler screening program for children with sickle cell anemia Pediatr Blood Cancer 2008 50 818 821 10.1002/pbc.21430 18085672

[B39] Vichinsky E Butensky E Fung E Hudes M Theil E Ferrell L Williams R Louie L Lee PD Harmatz P Comparison of organ dysfunction in transfused patients with SCD or b Thalassemia Am J Hematol 2005 80 70 74 10.1002/ajh.20402 16138345

[B40] Wood JC Tyszka JM Carson S Nelson MD Coates TD Myocardial iron loading in transfusion-dependent thalassemia and sickle cell disease Blood 2004 103 1934 1936 10.1182/blood-2003-06-1919 14630822

[B41] Wood JC Cardiac iron across different transfusion-dependent diseases Blood Rev 2008 22 2 S14 S21 19059052 10.1016/S0268-960X(08)70004-3 PMC2896332

[B42] Fung EB Harmatz PR Lee PD Milet M Bellevue R Jeng MR Kalinyak KA Hudes M Bhatia S Vichinsky EP Multi-centre study of iron overload research group: increased prevalence of iron-overload associated endocrinopathy in thalassaemia versus sickle-cell disease Br J Haemat 2006 135 574 582 10.1111/j.1365-2141.2006.06332.x 17054676

[B43] Fung EB Harmatz PR Milet M Coates TD Thompson AA Ranalli M Mignaca R Scher C Giardina P Robertson S Neumayr L Vichinsky EP Multi-center iron overload study group:fracture prevalence and relationship to endocrinopathy in iron overloaded patients with sickle cell disease and thalassemia Bone 2008 43 162 168 10.1016/j.bone.2008.03.003 18430624 PMC2500183

[B44] Cohen AR Martin MB Iron chelation therapy in sickle cell disease Semin Hematol 2001 38 suppl 1 69 72 11206964 10.1016/s0037-1963(01)90062-9

[B45] Silliman CC Peterson VM Mellman DL Dixon DJ Hambidge KM Lane PA Iron chelation by deferoxamine in sickle cell patients with severe transfusion-induced hemosiderosis: a randomized, double-blind study of the dose–response relationship J Lab Clin Med 1993 122 48 54 8320490

[B46] Lucania G Vitrano A Filosa A Maggio A Chelation treatment in sickle-cell-anaemia: much ado about nothing? Br J Haematol 2011 154 545 555 10.1111/j.1365-2141.2011.08769.x 21707578

[B47] Vichinsky E Onyekwere O Porter J Swerdlow P Eckman J Lane P Files B Hassell K Kelly P Wilson F Bernaudin F Forni GL Okpala I Ressayre-Djaffer C Alberti D Holland J Marks P Fung E Fischer R Mueller BU Coates T Deferasirox in sickle cell investigators: a randomized comparison of Deferasirox versus Deferoxamine for the treatment of transfusional iron overload in sickle cell disease Br J Haematol 2007 136 501 508 10.1111/j.1365-2141.2006.06455.x 17233848 PMC1974786

[B48] Vichinsky E Bernaudin F Forni GL Gardner R Hassell K Heeney MM Inusa B Kutlar A Lane P Mathias L Porter J Tebbi C Wilson F Griffel L Deng W Giannone V Coates T Long-term safety and efficacy of deferasirox (Exjade) for up to 5 years in transfusional iron-overloaded patients with sickle cell disease Br J Haematol 2011 154 387 397 10.1111/j.1365-2141.2011.08720.x 21592110 PMC3170481

[B49] Vichinsky E Pakbaz Z Onyekwere O Porter J Swerdlow P Coates T Lane P Files B Mueller BU Coïc L Forni GL Fischer R Marks P Rofail D Abetz L Baladi JF Patient-reported outcomes of deferasirox (Exjade, ICL670) versus deferoxamine in sickle cell disease patients with transfusional hemosiderosis. Substudy of a randomized open-label phase II trial Acta Haematol 2008 119 133 141 10.1159/000125550 18408362

[B50] Kinney TR Helms RW O’Branski EE Ohene Frempong K Wang W Daeschner C Vichinsky E Redding Lallinger R Gee B Platt OS Ware RE Pediatric Hydroxyurea Group Safety of hydroxyurea in children with sickle cell anemia: results of the HUG,KIDS study, a phase I/II trial Blood 1999 94 1550 1554 10477679

[B51] Wang WC Ware RE Miller ST Iyer RV Casella JF Minniti CP Rana S Thornburg CD Rogers ZR Kalpatthi RV Barredo JC Brown RC Sarnaik SA Howard TH Wynn LW Kutlar A Armstrong FD Files BA Goldsmith JC Waclawiw MA Huang X Thompson BW BABY HUG investigators Hydroxycarbamide in very young children with sickle,cell anaemia: a multicentre, randomised, controlled trial (BABY HUG) Lancet 2011 377 1663 1672 10.1016/S0140-6736(11)60355-3 21571150 PMC3133619

[B52] Hankins JS Helton KJ McCarville MB Li CS Wang WC Ware RE Preservation of spleen and brain function in children with sickle cell anemia treated with hydroxyurea Pediatr Blood Cancer 2007 50 293 297 17554794 10.1002/pbc.21271

[B53] Ferster A Vermylen C Cornu G Buyse M Corazza F Devalck C Fondu P Toppet M Sariban E Hydroxyurea for treatment of severe sickle cell anemia: a pediatric clinical trial Blood 1996 88 1960 1964 8822914

[B54] Hoots WK Shurin SB Future directions of sickle cell disease research: the NIH perspective Pediatr Blood Cancer 2012 59 353 357 10.1002/pbc.24180 22517801 PMC3374062

[B55] Berry JG Toomey SL Zaslavsky AM Jha AK Nakamura MM Klein DJ Feng JY Shulman S Chiang VW Kaplan W Hall M Schuster MA Pediatric readmission prevalence and variability across hospitals JAMA 2013 309 372 380 10.1001/jama.2012.188351 23340639 PMC3640861

[B56] Vichinsky EP Comprehensive care in sickle cell disease: its impact on morbidity and mortality Semin Hematol 1991 28 220 226 1887248

[B57] Yang YM Shah AK Watson M Mankad VN Comparison of costs to the health sector of comprehensive and episodic health care for sickle cell disease patients Public Health Rep 1995 110 80 86 7838948 PMC1382078

[B58] Okpala I Thomas V Westerdale N Jegede T Raj K Daley S Costello-Binger H Mullen J Rochester-Peart C Helps S Tulloch E Akpala M Dick M Bewley S Davies M Abbs I The comprehensiveness care of sickle cell disease Eur J Haematol 2002 68 157 162 10.1034/j.1600-0609.2002.01523.x 12068796

[B59] Amendah DD Mvundura M Kavanagh PL Sprinz PG Grosse SD Sickle cell disease-related pediatric medical expenditures in the U.S Am J Prev Med 2010 38 4 S550 S556 10.1016/j.amepre.2010.01.004 20331957

[B60] Kauf TL Coates TD Huazhi L Mody-Patel N Hartzema AG The cost of health care for children and adults with sickle cell disease Am J Hematol 2009 84 6 323 327 10.1002/ajh.21408 19358302

[B61] Colombatti R Montanaro M Guasti F Rampazzo P Meneghetti G Giordan M Basso G Sainati L Comprehensive care for sickle cell disease immigrant patients: a reproducible model achieving high adherence to minimum standards of care Pediatr Blood Cancer 2012 59 7 1275 1279 10.1002/pbc.24110 22359409

